# Modern concepts in facial nerve reconstruction

**DOI:** 10.1186/1746-160X-6-25

**Published:** 2010-11-01

**Authors:** Gerd F Volk, Mira Pantel, Orlando Guntinas-Lichius

**Affiliations:** 1 Department of Otorhinolarnygology, University Jena, Lessingstrasse 2, D-07740 Jena, Germany

## Abstract

**Background:**

Reconstructive surgery of the facial nerve is not daily routine for most head and neck surgeons. The published experience on strategies to ensure optimal functional results for the patients are based on small case series with a large variety of surgical techniques. On this background it is worthwhile to develop a standardized approach for diagnosis and treatment of patients asking for facial rehabilitation.

**Conclusion:**

A standardized approach is feasible: Patients with chronic facial palsy first need an exact classification of the palsy's aetiology. A step-by-step clinical examination, if necessary MRI imaging and electromyographic examination allow a classification of the palsy's aetiology as well as the determination of the severity of the palsy and the functional deficits. Considering the patient's desire, age and life expectancy, an individual surgical concept is applicable using three main approaches: a) early extratemporal reconstruction, b) early reconstruction of proximal lesions if extratemporal reconstruction is not possible, c) late reconstruction or in cases of congenital palsy. Twelve to 24 months after the last step of surgical reconstruction a standardized evaluation of the therapeutic results is recommended to evaluate the necessity for adjuvant surgical procedures or other adjuvant procedures, e.g. botulinum toxin application. Up to now controlled trials on the value of physiotherapy and other adjuvant measures are missing to give recommendation for optimal application of adjuvant therapies.

## Introduction

Although peripheral facial palsy is the most common pathology of the cranial nerves with an incidence ranging from 20 to 30 cases per 100.000 people per year, only a minority of the patients need a surgical treatment. During the acute phase of the palsy the indication for surgery is less dependent on the aetiology, but more on the individual chance of spontaneous and good functional recovery. In the chronic phase, surgery may be indicated in patients without or with unsatisfactory recovery, and in patients with defective healing. The appointed causes are viral infections such as reactivation of latent herpesvirus infection, trauma, iatrogenic injury, inflammatory affections of the middle ear, metabolic diseases and tumours affecting the facial nerve.

With 60% to 75% the major cause for facial palsy is idiopathic paralysis or Bell's palsy. 70% to 90% of patient with Bell's palsy recover completely, depending of an early start of steroid medication [1]. In contrast, in Ramsay-Hunt-Syndrome caused by reactivation of herpes zoster, the probability of complete recovery drops to 50%. Patient and treating physician should be aware, that many patients will need conservative and/or surgical treatment later on for defective healing.

Cholesteatoma of the middle ear and schwannomas of the facial or the vestibular nerve are less common causes of facial palsy, either by direct affection or iatrogenically during ear, parotid or skull base surgery. Here, as well as in trauma cases, mainly caused by temporal bone fractures or facial injuries due to traffic accidents or capital crimes, immediate or early surgical reconstruction might be indicated [2]. Indication for surgery is depending on the severity of the nerve lesion, i.e. blunt trauma leading to non-degenerative neuropraxia will not need surgical reconstruction, whereas disruption leading to degenerative neurotmesis will need surgery. Finally, any tumour in the course of the facial nerve from the brainstem to the periphery can cause facial palsy or surgical treatment of the tumour might be the reason for facial palsy. In such circumstances, typically surgery of the primary disease is combined with surgical reconstruction of the facial nerve [3].

## Definitions and classification

The term facial palsy summarizes incomplete loss (paresis) as well as complete loss (paralysis) of facial nerve function. The distinction is very important as the indication for surgical reconstruction in patients with incomplete facial palsy has to be proven much more critically. On the other hand, reconstruction in case of a complete functional deficit is more complex. Permanent facial palsy and non-transient functional deficits are the main indication for surgical reconstruction of facial nerve function.

Depending on the localisation of the lesion site, peripheral facial nerve lesion is separated from central facial nerve lesion: in peripheral palsy the facial nerve fibres or the motoneurons in the brainstem nucleus are damaged. In contrast, the lesion site in central palsy is located central to the nucleus (supranuclear lesion) in the course of the corticonuclear tract. The head and neck surgeon is mostly confronted with patients with peripheral nerve lesion. But sometimes the exact localisation of the lesion might be unclear, for instance in patients after brainstem astrocytoma surgery. The type of palsy must be clarified in front of reconstruction surgery as any kind of direct facial nerve reconstruction is not effective in patients with central palsy.

From the functional point of view two different situations have to be distinguished: First, patients without any sign of facial nerve regeneration due to complete hindrance of re-sprouting of the axons proximal to the lesion site are candidates. Second, patients who have developed spontaneous axonal sprouting but a functionally hindering defective healing not compensated by central brain plasticity are also candidates for surgical rehabilitation. Defective healing without spontaneous regeneration is impossible. The most important clinical signs of facial nerve defective healing are: a) dyskinesia, i.e. abnormal mimic movements during voluntary action, b) synkinesia, i.e. involuntary synchronous mimic movements while the patient is performing another voluntary movement, and c) autoparalytic syndrome as a special form of synkinesia characterized by synkinetic activity of antagonistic muscles. Synchronous antagonistic movements are detectable using electromyography but the clinical result is a decreased or unseeable muscle activity of the intended mimic movement. Dyskinesia and synkinesia can lead to d) hyperkinesia, i.e. abnormal and much stronger mimic movement than physiologically used.

An exact classification of the individual facial palsy due to the above mentioned criteria is mandatory prior to surgical decision making. In addition, the mimic musculature itself, the cerebral cortex and the other cranial nerves have to be examined for pathologies. Westin and Zuker have developed a simple and clear classification [4]. We recommend classifying each patient to our modified version of this classification directly leading to the optimal reconstruction strategy for the individual situation (Table 1).

**Table 1:** Classification of facial palsy and guidelines for their surgical reanimation (modified after [4])

Classification	Comments
A. Congenital	
A.1 syndromal A.2 non-syndromal	Mostly nerve plasty not possible; cortical deficits hinder additional mimic and physical training.
B. Acquired	
B.1 traumatic B.1.1 extracranial B.1.2 intracranial	Trauma: Exact localisation of lesion site mandatory. Acute nerve reconstruction only superior to conservative treatment in case of complete palsy.
B.2 tumourous B.2.1 extracranial B.2.1.1 benign	Tumour: Prognosis quoad vitam must be considered: prefer fast rehabilitation techniques.
B.2.1.2 malignant	
B.2.2 intracranial B.2.2.1 benign B.2.2.2 malignant	Intracranial: Reconstruction strategy without co-adaptation of the proximal facial nerve stump often the better choice.
B.3 infectious B.3.1 acute B.3.2 chronic	Infectious: Causal therapy in front, wait for reconstruction surgery after complete healing and look on remaining deficits.
B.4 neuromuscular B.4.1 Endplate region B.4.2 ganglional B.4.3 axonal	Neuromuscular: Domain of conservative neurologic treatment.

## Step-by-step preoperative evaluation

Intention of surgical reconstruction is to restore the function of the mimic musculature as optimal as possible. Under ideal circumstances this would be restoration of the resting tone of all mimic muscles and restoration of frontal frowning with lifting of the eye brow, closure of the eye, a symmetric nasolabial fold and the ability to smile nearly symmetrically. In patients with acute palsy a standardized clinical examination including analysis of voluntary movements (frowning, eye closure, nose wrinkling, showing the teeth, dropping of the angle of the mouth, pursing the lips) amended by electromyographic (EMG) evaluation is able to detect, which peripheral nerve branches and target muscles are affected or if the complete peripheral nerve is paralysed.

### Important role of EMG examination

**Figure 1:**
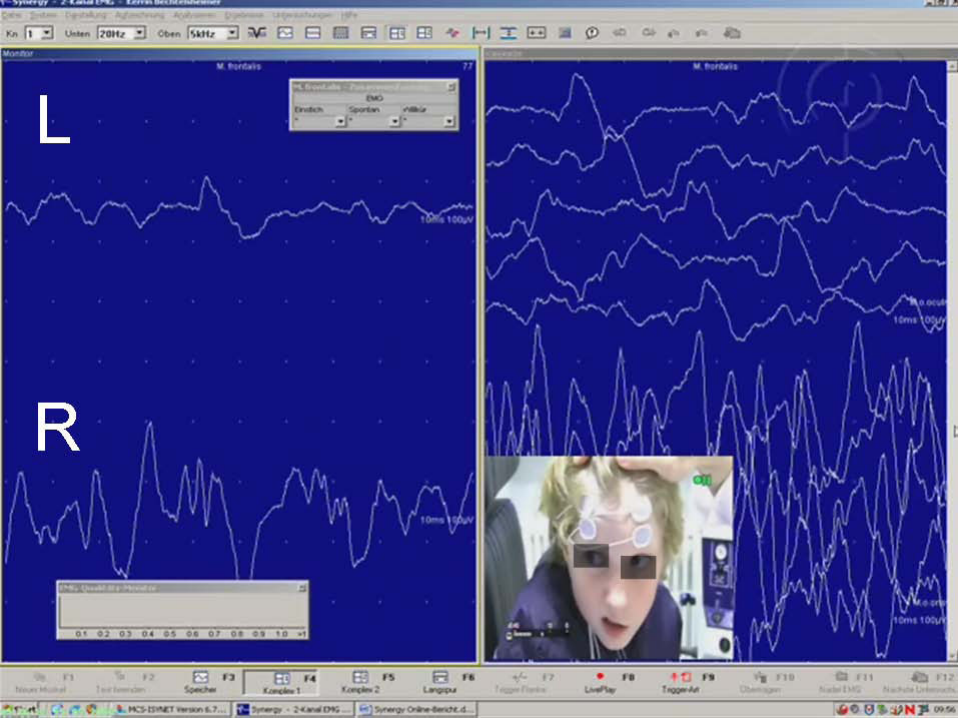
**Eletromyographic (EMG) analysis of a child with left side facial palsy after brainstem surgery**. Proof of complete loss of voluntary activity in left frontalis muscle (l) in comparison the healthy right side (r).

EMG plays a central role in the evaluation of the patient (Figure 1). Muscular damage leads to alterations of the insertion potentials during needle EMG. EMG allows a prognosis on the probability of spontaneous healing [5]. In congenital palsy or in chronic palsy EMG allows an assessment, if musculature (still) is existing and to what degree and in which regions of the face spontaneous regeneration with defective healing took place. In lesions proximal to the stylomastoid foramen disturbance of the lacrimal function and taste, or hyperacusis can be observed. In patients with regeneration and defective healing the clinical examination together with EMG allow the physician to evaluate the severity of dyskinesia, synkinesia, and autoparalytic syndrome [6].

### Magnetic resonance imaging

Magnetic resonance imaging (MRI) is preferred method of choice in order to localize a lesion of the facial nerve in the brainstem, the cerebellopontine angle and in the intratemporal course of the nerve [7]. MRI is much more accurate than classical topodiagnostic methods like Schirmer's test, stapedial reflex test, and taste function testing [8]. MRI also helps to evaluate the vitality of the mimic musculature in cases with long-term denervation. Muscle atrophy and fibrosis leads to an asymmetry of muscle volume in relation to the healthy side visible in MRI [9]. Such detailed analysis accounting for the patient's wishes and the life-expectancy of a comorbid patient should lead in an individual concept for the surgical rehabilitation of each patient.

## Selection of the optimal surgical concept for the individual patient

Basis for the selection of the rehabilitation technique of choice are the lesion site and the duration of palsy. Using these two parameters all surgical rehabilitation techniques can be divided in three categories (Table 2): a) early extratemporal reconstruction, b) early reconstruction in case of proximal lesion or impossibility of direct extratemporal reconstruction, and c) delayed or late reconstruction or congenital facial palsy.

**Table 2:** Plan by stages for facial reanimation (Modified after. [35])

Surgical method	Comments
A. Early reconstruction of extratemporal lesion	
Step I:	
A.1 Primary direct nerve suture	
A.2 Interpositional graft	
A.3 Upper lid weight	A.3. lid weight better than tarsorrhaphy
Step II:	
A.4 Adjuvant measures	
B. Early up to delayed reconstruction of proximal lesion or impossibility to use reconstruction A (see above)	
Step I:	
B.1 Hypoglossal-facial jump anastomosis	B.1 better than classical hypoglossal-facial anastomosis
B.2 Upper lid weight	
B.3 Cross-face nerve suture	
B.4 Temporalis muscle transfer	B.4 better than masseter muscle transfer
B.5 Digastric muscle transfer	
B.6 Sling plasty	
Step II:	
B.7 Cross-face nerve suture	
B.8 Eye brow lift	B.8. in case of brow ptosis
B.9 Rhinoplasty	B.9 in case of nasal asymmetry
B.10 Rhytidectomy	B. 10 in case of cheek or chin ptosis
B.11 Botulinum toxin, Myectomies	
C. Late reconstruction or congenital disease	
Step I:	
Mimic musculature existing:	
C.1 Hypoglossal-facial jump anastomosis	C.1 Hypoglossal nerve: better than any other donor nerve
C.2 Upper lid weight	
C.3 Cross-face nerve suture	
Mimic musculature not existing, but nerve supply existing:	
C.4 Microvascular muscle transfer	C.4 Best choice for congenital lesions
C.5 Temporalis muscle transfer	
Mimic musculature not existing, and nerve supply not existing:	
C.6 Sling plasty	C. 6 Use palmaris longus tendon or fascia lata
Step II:	
C.7 Eye brow lift	
C.8 Rhinoplasty	
C.9 Rhytidectomy	
C.10 Botulinumtoxin, Myectomies	C.10 Correction of defective healing or facial asymmetry on lesioned and healthy side

Early reconstruction means reconstruction within the first two months after lesion. In such a situation any nerve reconstruction will result in best possible functional recovery. Late reconstruction includes any repair 12 to 18 months after onset of the palsy. At this long denervation time irreversible atrophy and fibrosis has arisen if no regeneration occurred. Alternatively, if spontaneous but functionally insufficient regeneration emerged, defective healing has reached its final stage. Patients in-between these categories, i.e. a denervation time more than two months but less than twelve months, are difficult to categorize and must be considered individually after complete diagnostic examination.

## Early extratemporal facial nerve reconstruction

In patients with traumatic facial nerve lesion (most frequently intratemporally by temporal bone fracture or extratemporally due to acts of violence) or after malignant tumour resection (for instance in case of parotid cancer) primary facial nerve suture should be performed as fast as possible. In tumour patients it should be done directly in the same session with tumour resection to get the best results [3]. On the other hand, a good preoperative assessment is extremely important especially in polytrauma cases. In such cases, assessment is often limited to imaging techniques, and judgement of severity of the nerve lesion due to inspection or exploration. Eventually, the recovery of consciousness or the therapy of life-threatening injuries has to be awaited.

### Direct facial-facial nerve suture

In the first two months after trauma the nerve stumps can normally be dissected without hindering scar formation and best possible functional results can be achieved [6]. A direct co-adaptation of the facial nerve stumps is only possible, if the stumps are sharp-edged, i.e. after direct trauma, immediately within 24 hours after onset of the lesion.

### Facial nerve interpositional graft

Later, when the nerve stumps have to be freshened or if a gap of more than 1 cm is observed, an interpositional graft is needed to guarantee a tension-free nerve suture [3].

Well-proven donor nerves are the greater auricular nerve and the sural nerve. The use of biodegradable nerve tubes as alloplastic alternative can not be recommended for regular use as to date only case reports on their application are published [10].

### Hypoglossal-facial-jump-nerve anastomosis

Particularly after tumor resection the extratemporal resection defect can be very large in size. In such a situation a combined approach makes sense: The upper face is reconstructed with the proximal facial nerve and the lower face with a hypoglossal-facial-jump-nerve anastomosis. The separated reanimation of upper and lower face offers the advantage of prevention of synkinesia between both areas [6].

### Upper lid loading

Because the first clinical signs of a successful regeneration do not occur before a time of six months and the finial results even needs twelve to 18 months, nerve suture is often combined with static reanimation of the eye closure using a upper lid weight [11,12]. If lid weight is not effective, the first alternative is a palpable spring. This surgery is typically performed by an ophthalmologist [13]. If the lower lid is suspended due to loss of facial tone, it is recommended to combine upper eye lid surgery with a lower lid plasty [14].

### Dynamic muscle transfer

An alternative technique for the restoration of eye closure is to use a dynamic temporalis muscle plasty [15]. In individual cases, it could be reasonable to reanimate the angle of the mouth with a dynamic muscle plasty, too. But the surgeon has to take care not to injure the very thin facial nerve branches entering the orbicularis oris muscle. If the patient wishes a very fast solution or if life expectancy is low, a dynamic muscle plasty can also be performed as a single procedure without nerve reconstruction. Here, the temporalis muscle or the masseter muscle is used for perioral reconstruction in combination with upper lid weight for eye restoration [16]. Informed consent is necessary that the geometrical vectors of this kind of muscle plasties are limited. Muscle plasties only allow a few restored movements. A digastric muscle plasty is indicated for restoration of the depressor of the corner of the mouth in cases of isolated palsy of the marginal mandibular branch or congenital aplasia of the depressor anguli oris muscle [17].

### Sling plasties

Even a dynamic muscle plasty can be technically impossible in cases of extended tumour surgery. As third choice static slings are part of the surgical arsenal. Slings allow restoration of the resting tone and improvement of facial asymmetry at rest in direction of the inserted sling. Autologic material like fascia lata or the tendon of the palmaris longus muscle is first choice in front of alloplastic material. Complications, especially wound healing problems, are seen more frequently with alloplastic material [18].

## Early reconstruction in case of intratemporal, more proximal lesion or facial nerve lesion or no possibility for extratemporal reconstruction

For lesion of the facial nerve proximal to the stylomastoid foramen, especially in lesions proximal to the tympanic segment, it has to be proven carefully if nerve reconstruction with the proximal facial nerve still is first choice, or if a cross-nerve suture should be chosen instead. If an intratemporal facial nerve reconstruction is planned, an entire graft leads to better functional results than a partial graft (with the idea to preserve remaining intact nerve fibres) [19].

In general, the functional results in case of proximal facial nerve lesions seem to be better after cross nerve suture using a new motor nerve source than a far proximal nerve graft [6]. Anyway, both methods are functionally better than any elaborate intratemporal re-routing or even an intra-extracranial re-routing.

### Role of hypoglossal-facial-jump-nerve anastomosis in this setting

**Figure 2:**
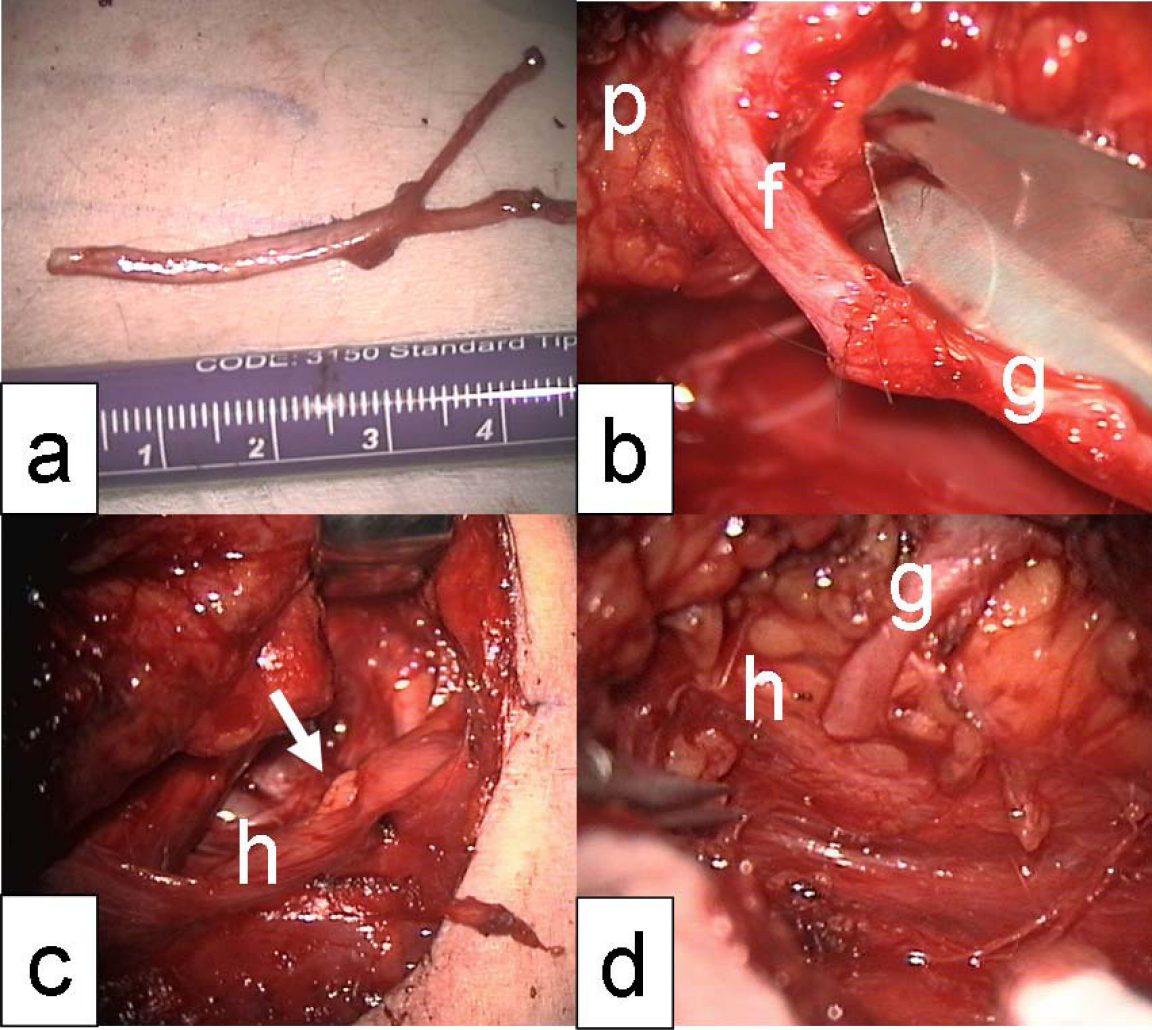
**Hypoglossal-facial jump nerve anastomosis**. a: Harvest of the greater auricular nerve as interpositional graft; b: End-to-end nerve suture of the graft (g) to the peripheral facial nerve (f); p = parotid gland; c: incision (arrow) of the hypoglossal nerve (h); d: end-to-side nerve suture between hypoglossal nerve (h) and the graft (g).

**Figure 3:**
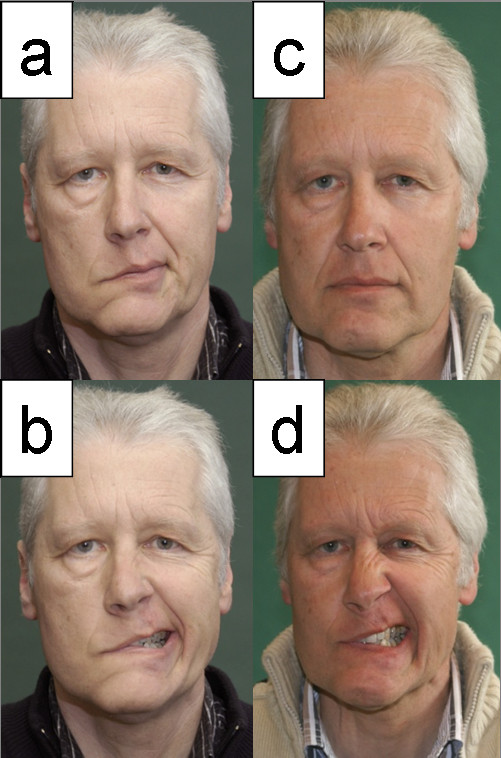
**a, b: Patient with complete facial palsy 5 months after vestibular schwannoma surgery; c, d: Same patient 2 years after hypoglossal-facial jump nerve anastomosis**. Pictures taken at rest (a, c) and during exposure of the teeth (b, d).

First choice for cross-nerve suture is the hypoglossal-facial jump nerve anastomosis (Figure 2 and 3). The classical type of hypoglossal-facial nerve anastomosis using the entire proximal hypoglossal nerve should be avoided nowadays. Classical hypoglossal-facial nerve anastomosis leads to unpleasant long-term sequelae, because the unilateral tongue atrophy produces permanent speech and swallowing problems. The hypoglossal-facial jump nerve anastomosis using only part of the hypoglossal nerve avoids tongue atrophy and the success rate is comparable to the classical type. Hyperkinesia, often seen after the classical technique, is avoided by the jump technique, because less nerve fibres regenerate to the periphery.

Several modifications of the hypoglossal-facial jump nerve anastomosis are described. Mostly used are a side-to-end nerve suture at the side of the proximal hypoglossal nerve and an end-to-end nerve suture to the distal facial nerve using a nerve graft in-between the hypoglossal and facial nerve. The hypoglossal nerve is incised to about 30%. Thereby, the nerve opens itself wedge-shaped to house the graft for the end-to-side nerve suture. Rarely, it is possible to bring together hypoglossal and facial nerve tensionless without using an interpositional graft. Other donor nerves for cross-nerve suture (motoric trigeminal nerve, accessory nerve, parts of the cervical plexus, ansa nervi hypoglossi) cause more morbidity in the donor region and show less satisfactory results [20].

### Cross-face facial nerve suture

The best alternative to hypoglossal-facial jump nerve anastomosis is a cross-face facial nerve suture: Peripheral facial nerve branches distal to the parotid gland are dissected on the contralateral healthy side. Even when electrostimulation is used to select two to four nerve branches to restore a selective symmetrical reinnervation of the ipsilateral lesioned side some additional palsy on the healthy side has to be accepted. To create a balance between these two aspects is difficult. The branches must be cut as distal as possible to minimize weakness on the healthy side. Long and several interpositional grafts are needed. Therefore, the suralis nerve is best choice. The suralis nerve is divided into several pieces. These pieces are pulled through the midface from the healthy to the lesioned side. The sural nerve grafts are sutured end-to-side to the facial nerve donor branches on the healthy side and end-to-end to selected peripheral facial nerve branches or to the main facial nerve trunk on the lesioned side[20].

Of course, depending on the individual situation, all kind of muscle plasties and sling procedures described above belong to the reanimation repertoire also in the situation of an early reconstruction in case of intratemporal lesion, more proximal facial nerve lesion or no possibility for extratemporal reconstruction.

## Late facial nerve reconstruction or congenital facial palsy

Beginning with a denervation time of six months or more, a strong vital motor nerve is needed to reanimate the mimic musculature. A hypoglossal-facial jump nerve anastomosis provides acceptable results up to about two years after onset of the lesion [6]. It should be kept in mind that the best results are reached within 2 months after onset of the lesion. A denervation time of six to twelve months guarantees at least satisfactory results. In case of longer denervation time the vitality of the mimic musculature has to be examined thoroughly. Age and comorbidity have influence on the velocity of muscle atrophy and fibrosis. In patients with a denervation time longer than two years, a nerve plasty without muscle transfer cannot be recommended on a regular basis. If a nerve reconstruction technique is chosen, the patient has to be informed that it takes six months on average before first signs of the muscle reinnervation are visible.

### Modifications of the cross-face facial nerve suture

If a cross-face facial nerve suture is chosen, even more time is needed because the grafts and therefore the distance to be reinnervated are much longer. To overcome this situation, the facial musculature of the lesioned side can be reanimated additionally by a so called babysitter procedure: Parallel to the cross-face surgery the facial musculature is reanimated by a hypoglossal-facial jump nerve anastomosis [21]. Recently, the babysitter procedure has also been described using the masseteric branch of the trigeminal nerve [22]. If the denervation time is longer than 6 months the proceeding fibrosis of the peripheral facial nerve could hinder the direct connection of the cross-face nerve suture to the target musculature. In such a situation, a different, two-step procedure is necessary: Nine to twelve months after the first step, when the nerve grafts are completely passed by the regrowing axons, the distal side of the grafts are connected to a free muscle transplant on the lesioned side (see below). A single step procedure, i.e. suture of the cross-face interpositional grafts and free muscle transfer at the same time in one surgical session, cannot be recommended as standard procedure as only limited data is published on this technique [23,24].

### Free muscle transfer

Free microvascular muscle transfer in combination with cross-face nerve suture is therapy of choice in patients with congenital facial nerve palsy (for instance in children with Moebius syndrome). Here, often the nerve and the mimic musculature do not exist [25]. The most frequent muscles used are the gracilis muscle and the pectoralis minor muscle [15,26]. In case of bilateral congenital palsy the reanimation of the free muscle transplant can be restored with bilateral hypoglossal-facial jump nerve anastomosis.

### Dynamic muscle transfer after long-term denervation

Especially in adult patients after tumor surgery, the use of dynamic muscle transfer (see above) is a good alternative to elaborate nerve reconstructions.

## Adjuvant measures

Twelve to 24 months have to be awaited for the first reanimation sign and later the complete reinnervation of the face after any kind of nerve surgery. Many patients need additional small surgery to correct smaller complaints due to the chronic palsy and the reanimation surgery. The patients should already be informed about this fact in front of any surgery during the planning phase.

### Botulinum toxin therapy

**Figure 4:**
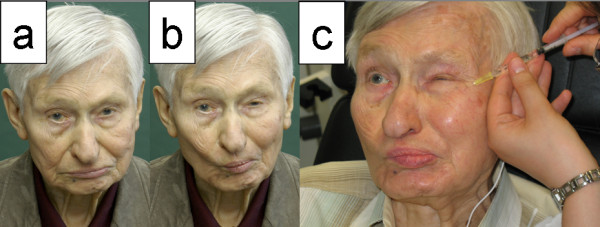
**Patient with oro-ocular synkinesia after severe Bell's palsy of left side; Pictures taken at rest (a) and with pursed mouth and involuntary synkinetic closure of the left eye (b)**. Treatment of the synkinesia with botulinum toxin injection into the orbicularis oculi muscle (c)

Dyskinesia and synkinesia as result of effective nerve regeneration can be reduced effectively by botulinum toxin injections (Figure 4) [27]. The reversibility of the botulinum toxin effect allows an individual adoption of necessary treatment. Since the introduction of botulinum toxin for this indication, definitive selective myectomies or neurectomies are no longer necessary. These irreversible and rough procedures should only be discussed if botulinum toxin is not effective. In facial areas with permanent weakened movements the asymmetry to the contralateral facial side is even amplified by overuse of the contralateral healthy side. In such a case, botulinum toxin can also be applied on the healthy side to reduce the muscle movements in the overused mimic areas. On the healthy side, botulinum toxin is most often used to reduce the function of the depressor anguli oris muscle [28].

### Mimic therapy and physical therapy

Mimic therapy should start at best when the first reinnervation signs are visible by EMG or are at least when reinnervation is clinically visible in the mimic musculature after nerve reanimation surgery. Before, mimic therapy only is frustrating for the patients, because it will not result in voluntary movements. In case of hypoglossal-facial jump nerve anastomosis, the training must focus first on intended tongue movements to induce facial mimic movement. The patient will learn which kind of intended tongue movements lead to which kind of facial movement. With time, the patient will move his face without thinking on tongue movements anymore.

Systematic controlled studies on the role of physical therapy and also on the role of electrostimulation therapy are lacking [29,30]. It is imaginable that physical therapy could help to reduce the degree of muscle atrophy in the first time after nerve suture to bridge the time before the regrowing axons have reached the mimic musculature. In patients with muscle transfer physical therapy could start after wound healing and help the patient to train the transferred muscle for his new function [31].

## Evaluation of the surgical results

Most clinical studies on the results of facial nerve reconstruction use (beside photographs) the House-Brackmann grading system, although this system was only developed to classify acute facial palsy. Assessment of defective healing is not part of this classification system. Therefore, other systems including the assessment of defective healing are more suitable for evaluation of the surgical results. Such systems are: Stennert Index, Sydney system or the Sunnybrook system [6,32,33]. Even better are objective observer-independent measurement tools like video-based semiquantitative measurement systems. But up to now, these system has not become part of clinical routine [15]. Beside the functional evaluation, the assessment should nowadays also include the measurement of quality of life after facial reconstruction surgery [34].

## Conclusion

**Figure 5:**
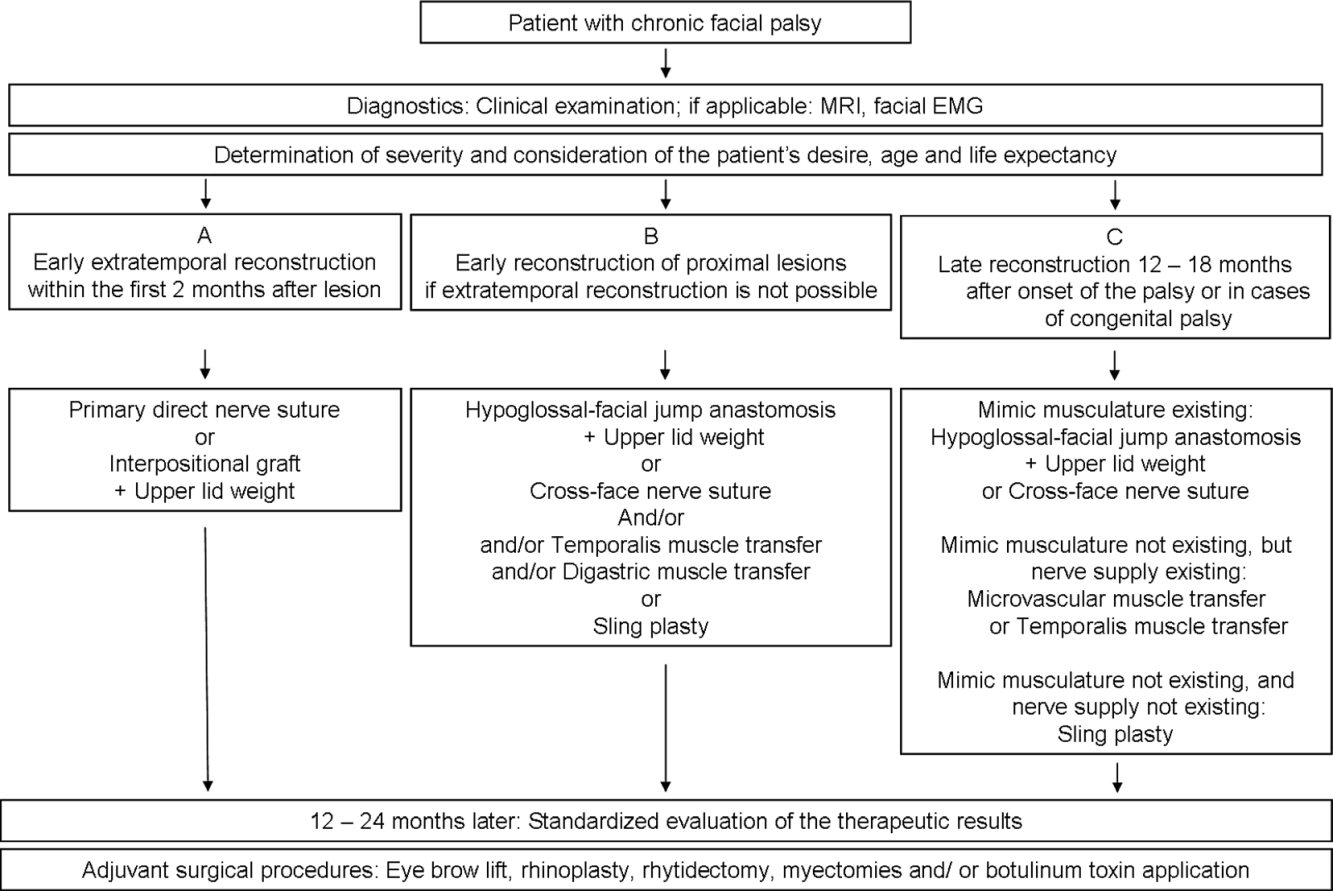
**Summarizing schematic algorithm of the different possibilities of facial nerve reconstruction**.

Head and neck surgeons faced with acute or chronic facial palsy demanding surgical repair need a broad spectrum of surgical tools in order to ensure optimal treatment of the patient. Following the diagnostic recommendations and the classification presented in this review may help to find the optimal strategy of modern facial nerve rehabilitation for the individual patient with severe facial palsy (Summary in Figure 5).

## Consent

It is stated that informed written consent was obtained for publication of the patients images.

## Abbreviations

EMG: electromyography; MRI: Magnetic resonance imaging.

## Authors' contributions

The authors issued the whole manuscript. All three authors have read and approved the final manuscript.

## Competing interests

The authors declare that they have no competing interests.
